# 34例成人系别模糊的急性白血病病例分析

**DOI:** 10.3760/cma.j.issn.0253-2727.2023.11.010

**Published:** 2023-11

**Authors:** 彩霞 裴, 茜 詹, 岑鸟 刘, 文 彭, 利 王, 林 刘, 渊杰 李, 毅 廖, 小华 罗

**Affiliations:** 1 重庆医科大学附属第一医院血液内科，重庆 400016 Department of Hematology, the First Affiliated Hospital of Chongqing Medical University, Chongqing 400016, China; 2 重庆医科大学附属第一医院临床分子医学检测中心，重庆 400016 The Center for Clinical Molecular Medical Detection, the First Affiliated Hospital of Chongqing Medical University, Chongqing 400016, China; 3 重庆市合川区人民医院血液内科，重庆 401519 Department of Hematology, Hechuan People's Hospital of Chongqing, Chongqing 401519, China; 4 重庆市第四人民医院血液内科，重庆 400014 Department of Hematology, the Fourth Hospital of Chongqing, Chongqing 400014, China

系别模糊急性白血病（Acute leukemia of ambiguous lineage，ALAL）是由免疫分型定义的高危急性白血病，其中急性混合细胞白血病（MPAL）患儿和成人的存活率分别为47％～75％和20％～40％，关于最佳治疗方案尚无共识[1]–[2]。1995年Catovsky建立了混合白血病诊断标准，4年后由欧洲白血病免疫表型小组（EGIL）提出成为白血病免疫分型诊断基础。诊断MPAL时，每个系别得分至少2.5分，根据EGIL评分系统可分为T/髓（T/M）、B/髓（B/M）或T/B/髓（T/B/M）[3]。根据2022年第五版WHO造血淋巴肿瘤新分类（WHO-HAEM5标准）ALAL分为具有明确遗传学异常的ALAL（包括MPAL伴BCR-ABL融合基因、MPAL伴KMT2A重排、ALAL伴BCL11B基因重排、MPAL伴ZNF384基因重排）和根据免疫表型定义的ALAL［包括B/M表型的MPAL、T/M表型的MPAL、罕见型的MPAL、不能分类的ALAL以及急性未分化型白血病（AUL）］[4]。我们采用EGIL积分系统和WHO-HAEM5标准，回顾性分析了34例ALAL患者的临床特征、细胞形态、免疫表型、细胞遗传学、分子生物学以及治疗和预后，现汇报如下。

## 病例与方法

1. 研究对象：纳入2014年1月1日年至2022年6月30日就诊于我院血液科的34例确诊ALAL患者，采用常规方法进行细胞形态学、免疫学以及分子生物学和细胞遗传学检查。ALAL诊断有赖于免疫分型，流式细胞术是确诊的首选方法[5]，本中心病例主要根据EGIL积分系统和WHO-HAEM5标准来诊断ALAL。

2. 治疗方案：初次诱导缓解方案包括淋系方案VDLP（长春地辛、多柔比星、培门冬酶、地塞米松），髓系方案IA（去甲氧柔红霉素、阿糖胞苷）、DA（柔红霉素、阿糖胞苷），以及髓淋系兼顾方案DA+VP（柔红霉素、阿糖胞苷、长春地辛、地塞米松）。对于Ph^+^ MPAL患者，诱导化疗联合酪氨酸激酶抑制剂（TKI）。

3. 疗效标准及研究终点：根据《血液病诊断及疗效标准》评估疗效，通过查阅临床资料或者电话随访，所有患者随访至2023年2月26日。总生存（OS）期为初次诊断至随访时间或任何原因导致死亡的时间。

4. 统计学处理：采用SPSS 20.0进行统计学处理。数据以中位数、均值或百分率表示，率的比较采用Fisher确切检验或*χ*^2^检验，定量资料采用Kruskal-Wallis检验或方差分析，Kaplan-Meier法进行生存分析，生存率的比较采用Log-rank检验，以*P*<0.05 为差异有统计学意义。

## 结果

1. 患者基本资料：本数据来源于重庆医科大学附属第一医院1 367例急性白血病患者，其中ALAL 34例，约占全部急性白血病的2.5％。ALAL患者男20例（58.8％），女14例（41.2％），中位年龄47（15～76）岁，均为汉族。32例ALAL患者收集到完整外周血常规结果资料，25例（78.1％）患者外周血幼稚细胞比例中位数76％（3％～94％）。初诊时患者外周血中位WBC为4.78（0.24～261）×10^9^/L，WBC>50×10^9^/L者7例，其中5例为Ph阳性MPAL，白细胞计数明显高于T/M、B/M两组；中位HGB 71（42～197）g/L，PLT 122（5～394）×10^9^/L，ALAL患者表现为两系或者三系减少。ALAL患者就诊时临床表现发热9例（26.5％），淋巴结肿大20例（58.8％），脾大12例（35.3％，其中2例为Ph阳性MPAL），乏力7例（20.6％），咳嗽6例（17.6％），腹泻4例（11.8％），出血5例（14.7％），2例（5.9％）存在中枢神经系统白血病。ALAL患者经临床诊断T/M-MPAL 17例（50.0％），B/M-MPAL 11例（32.4％），MPAL伴BCR-ABL（Ph^+^ MPAL）5例（14.7％），AUL 1例（2.9％）。不同ALAL亚组间患者初诊时临床特征详见表1。

**表1:** 34例系别模糊急性白血病患者临床特征

特征	总体（34例）	Ph^+^ MPAL（5例）	T/M-MPAL（17例）	B/M-MPAL（11例）	AUL（1例）
年龄[岁，*M*（范围）]	51（15~76）	57（32~65）	27（15~76）	52（24~66）	70
性别[例（%）]					
男	17（50.0）	1（20.0）	11（64.7）	4（36.4）	1（100）
女	17（50.0）	4（80.0）	6（35.3）	7（63.6）	0（0）
HGB[g/L，*M*（范围）]	74（42~197）	105（90~119）	88（56~197）	72（42~100）	69
PLT[×10^9^/L，*M*（范围）]	80（5~394）	84（27~159）	122（10~257）	32（5~394）	325
WBC[×10^9^/L，*M*（范围）]	4.8（0.2~261.0）	181.0（58.4~261.0）	4.8（1.8~249.0）	4.6（0.2~33.0）	4.4
外周血幼稚细胞[%，*M*（范围）]	76.5（3~94）	76（63~87）	77（16~94）	36（3~93）	0

注 MPAL：急性混合细胞白血病；AUL：急性未分化型白血病

2. 骨髓细胞形态学：34例患者中33例有骨髓细胞形态结果，形态学诊断为AML 13例（39.4％），诊断为淋巴瘤/急性淋巴白血病11例（33.3％）；3例（9.1％）诊断为MPAL；余6例（18.2％）不能分类。33例患者中30例（90.9％）骨髓增生程度为明显或极度活跃。ALAL患者形态学显示原始细胞比例中位数为78％（7％～97％），27例（81.8％）患者骨髓原始细胞核仁明显。

3. 流式细胞术免疫分型：34例初诊ALAL病例，12例（35.3％）为双克隆，21例（61.8％）为双表型；34例患者中符合EGIL者31例，符合WHO标准者28例，同时符合EGIL和WHO标准者25例（73.5％）；符合EGIL积分31例，其中T/M-MPAL 16例（51.6％），B/M-MPAL 15例（48.4％）；符合WHO-HAEM5标准 28例，其中T/M-MPAL 11例（39.3％），B/M-MPAL 10例（35.7％），Ph^+^ MPAL 5例（17.9％），AUL 2例（7.1％）。原始细胞标志中，CD34^+^ 31例（91.2％），CD38^+^ 31例（91.2％），HLA-DR^+^ 28例（82.4％）。髓系标志中，CD117^+^ 30例（88.2％），CD13^+^ 30例（88.2％），CD33^+^ 28例（82.4％），cMPO^+^ 10例（29.4％），CD15^+^ 24例（70.6％），CD64^+^ 4例（11.8％），CD11c^+^ 11例（32.4％）。17例T/M-MPAL患者中，T系标志cCD3^+^ 17例（100％），CD7^+^ 17例（100％），CD2^+^ 9例（52.9％），TdT^+^ 8例（47.1％），CD5^+^ 6例（35.3％）；在10例B/M-MPAL患者中，B系标志cCD79a^+^ 9例（90％），CD22^+^ 8例（80％），CD19^+^ 9例（90％），CD20^+^ 1例（10％），TdT^+^ 6例（60％），CD10^+^ 5例（50％）。

4. 细胞遗传学：19例ALAL患者有完整染色体核型分析结果，正常核型10例（52.6％），异常核型9例（47.4％）。异常核型中，T/M-MPAL 4例，B/M-MPAL 3例，Ph^+^ MPAL 1例，AUL 1例；其中3例为复杂核型（异常核型>3种），1例伴有TP53突变；2例伴有染色体−7，2例伴有染色体−5，1例Ph^+^，染色体异常集中在2、6、7、8、9、10、11、12、17、20、22等（表2）。

5. 分子生物学：24例行43种融合基因检测。5例（20.8％）患者发生BCR-ABL基因融合，3例为BCR-ABL p190，2例为BCR-ABL p210；1例发生SET-CAN 融合。25例行预后基因检测，20例采用sangers测序法，其中FLT3-TKD突变4例（16％），RUNX1突变3例（12％），TP53突变2例（8％），DNMT3A突变2例（8％），NOTCH1、IDH1、IDH2、C-KIT17、EZH2突变各1例（4％）；5例采用二代测序方法，1例Ph^+^ MPAL患者发生DNMT3A、FLT3-ITD、ASXL1、NRAS多基因突变；4例T/M-MPAL患者多基因突变（表2）。

**表2:** 28例系别模糊急性白血病患者细胞遗传学和分子表型

例号	克隆性	临床诊断	细胞遗传学	分子表型
1	未分类	AUL	46XY,+10[1]/48idem,+11[1]/46,XY[18],+10	N
2	双表型	B/M	46,XX,t(2:5)(p13:q13),del(13)(q12q14)[1]	SET-CAN
3	双表型	B/M	46,XY[20]	/
4	双克隆	B/M	46,XY,+6,+8,−11,−12,+6~8mar[cp6]/46,XY[14]	N
5	双克隆	B/M	46,XY,del(20)(q12)[12]/46,idem,del(7)(q22)[3]/46,XY[5]/46,XX[5]	N
6	双克隆	B/M	/	CKIT17 D816V突变
7	双表型	B/M	46,XX[10]	TP53突变
8	双表型	B/M	46,XY[5]	N
9	双克隆	B/M	46,XX[5]	IDH2
10	双表型	Ph^+^	46,XY[5]	BCR-ABLp190
11	双克隆	Ph^+^	/	BCR-ABLp190
12	双克隆	Ph^+^	/	BCR-ABLp210
13	双克隆	Ph^+^	46,XX,t(?4;9;22)(q?33;q34;q11)[5]	BCR-ABLp190
14	双克隆	Ph^+^	/	BCR-ABLp210
				DNMT3A、FLT3-ITD、ASXL1、NRAS
15	双表型	T/M	46,XY[20]	N
16	双表型	T/M	46,XY[5]	N
17	双表型	T/M	46,XX	N
18	双表型	T/M	46,XX,del(4)(qx),t(5;10)(p15;q21)[8]	EZH2
19	双表型	T/M	/	FLT3(TKD)、TET2、RUNX1
20	双表型	T/M	46,XX,del(5)(q31)[13]/46,XX[5]	/
21	双表型	T/M	46,XY[20]	FLT3-TKD、DNMT3A、RUNX1
22	双表型	T/M	/	RUNX1、ASXL1、NOTCH11
23	双表型	T/M	45,XY,del(2)(q35),−5,add(12)(p11.2),−17,del(20)(q13.1),+mar[13]/45,idem,t(9;15)(q34;q22)[7]	TP53
24	双表型	T/M	/	/
25	双表型	T/M	/	CEBPA、GATA2、MUC16
26	双克隆	T/M	46XX,add(2)(p25),add(6)(p25),del(6)(q23),del(7)(q22)[20]	IDH1
27	双克隆	T/M	46，XY[20]	N
28	双克隆	T/M	/	FLT3-TKD、PHF6

注 N：未检测出融合基因和基因突变；/：未检测

6. 疗效评估与预后：34例ALAL患者中25例接受治疗，第1疗程化疗后完全缓解（CR）率36.0％（9/25），未缓解患者中5例放弃治疗，调整诱导化疗方案后，CR率达68.0％（17/25），其中T/M-MPAL 84.6％（11/13），Ph^+^ MPAL 66.7％（2/3）；B/M-MPAL37.5％（3/8），AUL 1例CR至今，无病生存5年。诱导化疗方案以淋系为主的CR率达60.0％（6/10），以髓系方案为主的CR率达75.0％（3/4），以髓淋兼顾方案的CR率35.0％（7/20）。17例诱导治疗达CR的患者，存活3例，5例发生感染放弃治疗，4例复发难治，5例失访。5例进行异基因造血干细胞移植的患者，2例生存至今，另外3例患者死亡。移植组中位OS期12个月，2年OS率达40％；化疗组中位OS期7个月，2年OS率为0。ALAL患者中位OS期8个月。

3例Ph^+^ MPAL患者中，1例使用VID+伊马替尼方案化疗后达CR，后行造血干细胞移植，移植后发生肝窦阻塞综合征（SOS）死亡；1例采用髓淋兼顾方案+达沙替尼方案行7个疗程化疗，均达缓解状态，第7疗程化疗后发出现粒细胞缺乏伴感染后拒绝继续治疗出院；1例首次行髓淋兼顾方案化疗，未用TKI，化疗后达CR，再次入院后显示复发，因白细胞淤滞导致脑血管意外。

在进行流式细胞术免疫微小残留病（MRD）监测中，选择有连续治疗经过和免疫表型的2例B/M-MAPL患者，收集和分析患者免疫微残留监测B系和髓系的变化，见图1。

（1）患者A细胞形态学诊断AML，流式细胞术免疫分型检测为B/M-MPAL（双克隆）；首次诱导化疗采用髓淋兼顾方案，化疗后骨髓形态检测未达CR，随后给予髓系方案化疗，骨髓形态检测达部分缓解，流式细胞术MRD检测显示异常B系原始细胞百分比（1.70％）首次低于髓系原始细胞（3.42％）。第3个疗程化疗继续使用髓系方案，形态学达CR，流式细胞术MRD检测显示异常髓系原始细胞1.16％，异常B系原始细胞0.19％。第4个疗程化疗采用淋系方案，流式细胞术MRD检测显示异常髓系原始细胞0.02％。第5个疗程淋系方案化疗后流式细胞术MRD阴性，遂行异基因造血干细胞移植，患者存活至今，无病生存2年（图1A）。

（2）患者B首次诱导化疗采用以髓系为主方案无效后改为淋系方案，未达CR，流式细胞术检测新发现占比19％幼稚单核细胞，后行淋系方案化疗，形态学达CR；继续行淋系方案化疗后流式细胞术MRD检测阴性。因患者及家属拒绝造血干细胞移植，后继续淋系方案化疗，流式细胞术MRD检测发现6％异常幼稚单核细胞。入院前复查骨髓显示白血病复发，采用髓系方案化疗后未缓解且合并严重感染，患者放弃治疗出院（图1B）。

**图1:**
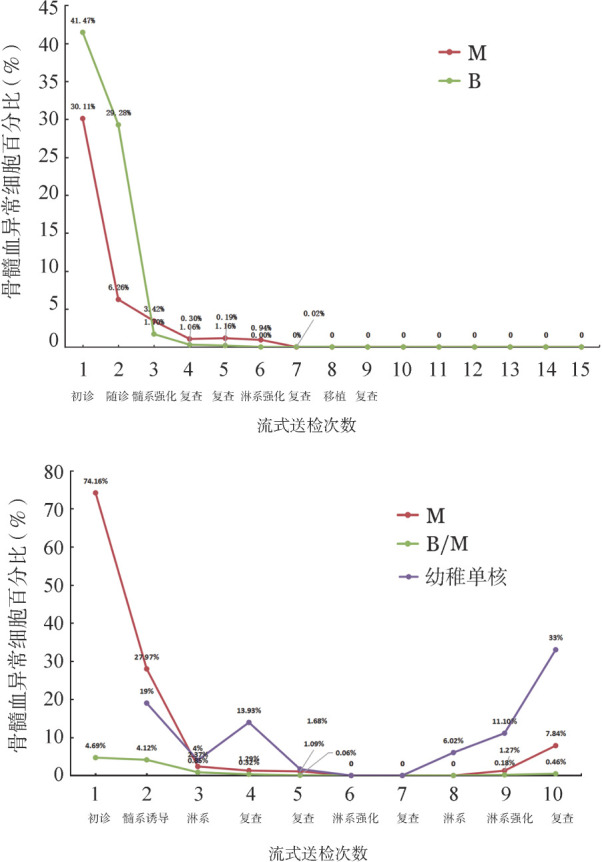
两例B/M表型急性混合细胞白血病患者免疫微残留在治疗过程中的动态变化 A 患者A； B 患者B

## 讨论

ALAL是一类罕见的异质性急性白血病，具有复杂的表型和遗传基础。本研究发现，ALAL发病率约占同期成人急性白血病的2.5％，符合全球发病率2％～5％[1]。ALAL初诊患者临床表现和常见急性白血病类似[2]。从血象来看，ALAL患者普遍三系或者两系降低，外周血出现不同比例的幼稚细胞。诊断Ph^+^MPAL时，需与慢性髓性白血病（CML）急变做鉴别诊断[1]，本研究中MPAL患者起病急，均在半个月以内发现，且没有CML病史，预后差[4]。

在诊断ALAL时，本研究显示EGIL积分系统与WHO-HAEM5标准相比更具有包容性，但EGIL积分系统仅依靠流式细胞术免疫积分，评估结果简单；而WHO-HAEM5标准[4]结合MICM将ALAL分为具有明确遗传异常的ALAL和具有免疫表型定义的ALAL，新增了新的遗传亚型以及对MPAL的谱系分配标准进行了改进，更强调荧光强度和抗原组合的重要性，从而更精细地对ALAL进行分类。既往研究表明，不符合WHO 2008/2016年分类的ALAL患者预后更差[6]–[7]，本研究中，不符合WHO-HAEM5标准的患者也表现出预后差的现象，但也和病例数较少有关。由于此类疾病的复杂性，目前诊断ALAL疾病仍需同时结合EGIL积分系统和WHO-HAEM5标准。

CD34是造血干细胞的特征性标志，在本研究ALAL患者中CD34表达率高达80％以上，高于AML和ALL中表达率[8]。CD7在Ph^+^ MPAL和B/M-MPAL的表达率分别为66.7％和50％，远远高于AML（20％～30％）[9]。Gupta等[10]研究表明 CD7 表达可能与骨髓发育早期干细胞的起源有关以及和总体预后不良有关。

ALAL患者基因突变涉及多种功能，包括染色质修饰（ASXL1）、DNA甲基化（DNMT3A、IDH和TET2）、肿瘤抑制因子（TP53和WT1）、转录因子（NOTCH1、RUNX1和GATA1）和激活信号传导（FLT3、EGFR、NRAS和JAK2）等，这些基因主要与造血谱系分化有关。另一研究证明了BCL11B可以上调表达cCD3^+^CD34 ^+^ HSPC中的T细胞分化基因表达程序，提出了T谱系表型完全由BCL11B活性决定[11]。此类疾病中，FLT3突变最常见（15％～30％），并提出了FLT3抑制剂可能在BCL11B失调的白血病中具有治疗相关性。与既往研究结果类似[12]–[13]，本组28例ALAL患者中75％存在核型异常和基因突变，33.3％患者为复杂核型，多例患者合并谱系分化相关的基因突变。

由于MPAL中可能同时存在不同表型或不同系列的恶性克隆，白血病细胞会通过改变转录程序分化为不同的谱系。本研究中2例MPAL患者骨髓中MRD动态变化可能反映了患者体内白血病优势克隆的改变。1例初诊时为B/M-MAPL，当时骨髓中并没有幼稚单核细胞，而在治疗过程中出现幼稚单核细胞后疾病迅速进展，肿瘤细胞的克隆演化可能与疾病的高复发性密切相关。研究表明AML细胞在复发时可能会获得一些新的突变，这些突变有助于克隆选择和导致化疗耐药性[14]。因此治愈这类疾病需根除新出现的克隆及所有克隆，实时关注免疫MRD结果以及相关基因表达变化以观察优势克隆，并及时调整方案可能对提高患者生存率有一定帮助。

虽然ALAL患者最佳治疗方案尚不清晰，但细胞分子遗传学的发展促进了这一疾病的分类，在2008 WHO分型中根据细胞遗传学加入了Ph^+^ MPAL类别，针对这一类患者可以加入TKI治疗[1]，联合TKI可明显提高患者CR率并延长无病生存期[15]。在本研究中，2例Ph^+^ MPAL患者首次诱导治疗使用TKI后均达CR。

除了Ph^+^ MPAL外，Andrews等[16]报道FLT3抑制剂在两例FLT3突变MPAL患者中获得疗效。Klocke等[17]报道BCL-2抑制剂维奈克拉联合去甲基化药物诱导治疗MPAL患者获缓解。CAR-T细胞免疫疗法已开始成功用于治疗MPAL [18]，有报道采用奥加伊妥珠单抗靶向CD22抗体治疗未分类急性白血病[19]。总之，ALAL化疗缓解率低，远期预后差，虽然初步取得一定治疗效果，但对该类疾病发病机制和治疗方案仍需要进一步研究。
